# DFRSeisNet: Fluctuation-Prior-Regularized Background Noise Attenuation for Seismic Signal Denoising

**DOI:** 10.3390/s26185938

**Published:** 2026-09-19

**Authors:** Fei Deng, Liang Pang, Shuang Wang, Wen Peng

**Affiliations:** 1 College of Computer Science and Cyber Security, Chengdu University of Technology, Chengdu 610059, China; dengfei@cdut.edu.cn; 2 Key Laboratory of Earth Exploration and Information Techniques of Ministry of Education, Chengdu University of Technology, Chengdu 610059, China; wangs@stu.cdut.edu.cn; 3 Research and Development Center, Bureau of Geophysical Prospecting, China National Petroleum Corporation, Zhuozhou 072751, China; pengwen_wt@cnpc.com.cn

**Keywords:** seismic denoising, deep learning, fluctuation prior, global consistency, patch-based processing

## Abstract

Seismic exploration has progressively expanded into urban fringe regions and areas with intensive human activities, where anthropogenic interference has increased markedly, aggravating the background noise problem and substantially affecting the accuracy of subsequent seismic data processing. Conventional denoising methods struggle to cope with such complex noise patterns and typically rely on manually designed parameter settings, which limits their ability to suppress complex background noise in real seismic data. With the rapid development of deep learning, various neural-network-based methods have been introduced for seismic data denoising. However, existing deep learning approaches are constrained by the high resolution of seismic data and limited computational resources and therefore commonly perform denoising on cropped data patches rather than on the complete seismic section. This limitation weakens the network’s capability to perceive global contextual information, degrading denoising performance and potentially introducing blocking artifacts. To address these issues, we propose a fluctuation-prior-regularized denoising framework that explicitly decomposes the complete seismic data denoising task into global and local denoising subtasks, enabling globally consistent denoising under complex conditions. To overcome the limitations of CNNs and Transformers, we adopt Retentive Networks Meet Vision Transformers (RMT) as the backbone for feature extraction in this work. And a fluctuation-prior-constrained local denoising mechanism is introduced, allowing local patches to indirectly capture global information. In addition, a grouped regularization strategy for global and local tasks is proposed, enabling both optimization tasks to better capture their respective task-specific characteristics. Experimental results on noisy shot gathers constructed from field records and on field-recorded background noise demonstrate that the proposed DFRSeisnet achieves superior denoising performance while effectively alleviating blocking artifacts.

## 1. Introduction

In recent years, as seismic exploration has increasingly expanded into urban and industrial areas [1,2,3], the level of background noise interference during data acquisition has risen notably. Background noise is complex in composition and diverse in origin, primarily including instrument noise [4,5], natural environmental noise [6], and noise generated by human activities [7]. Instrument noise typically arises from mechanical vibrations and poor sensor coupling [8]. Natural environmental noise is mainly wind-induced, exhibiting pronounced nonstationarity and randomness, with statistical properties that vary with time, location, and environmental conditions [9]. Noise from human activities often shares overlapping temporal, spatial, and frequency characteristics with useful seismic signals [10]. Owing to the diversity of noise types, their broad spectral coverage, and their strong randomness, background noise can severely degrade the reliability of subsequent imaging and interpretation. Therefore, effective suppression of background noise is a critical step in seismic data processing [11,12]. In this study, background noise refers to source-independent noise recorded during land seismic acquisition, including nonstationary environmental and anthropogenic components that may overlap with reflection signals in time, space, and frequency.

Current seismic data denoising methods can be broadly categorized into traditional filtering approaches and deep-learning-based approaches. Traditional filtering methods rely on preset parameters determined by data characteristics. For example, low-pass, high-pass, and median filtering suppress noise by exploiting differences in frequency-domain distributions [13]; Radon [14] and F–K [15] filtering attenuate noise based on differences in propagation direction; and wavelet [16] or curvelet [17] transforms remove noise using differences in spatial distribution. However, in background-noise suppression tasks, many noise components overlap heavily with the characteristics of useful seismic signals. Consequently, when background noise overlaps with reflection signals in frequency, apparent velocity, or local dip, purely filtering-based approaches may require careful parameterization and can attenuate weak reflection energy if the signal–noise separation assumptions are not satisfied [18,19]. In addition, these approaches typically depend strongly on parameter tuning, placing high demands on the operator and making them unsuitable for data severely contaminated by strong background noise [20,21].

Deep learning methods have achieved remarkable performance in seismic data denoising owing to their powerful feature extraction capabilities. At present, deep learning-based seismic denoising approaches can be broadly categorized into CNN-based and Transformer-based methods. Among CNN-based approaches, Fang et al. [22] proposed a novel unsupervised random-noise suppression method that directly trains the network on noisy target data without requiring noise-free labels. Ma et al. [23] incorporated the alternating direction method of multipliers (ADMM) into DnCNN to decompose seismic data for low-frequency desert seismic noise attenuation. Dong et al. [24] developed a multiscale spatial attention denoising network, termed MSSA-Net, which integrates multiscale feature extraction and spatial attention mechanisms to improve strong-noise suppression and weak-signal recovery. Among Transformer-based approaches, Wang et al. [25] proposed a Transformer-based self-supervised pretraining framework for complex seismic noise removal. Zhang et al. [26] introduced a Swin Transformer-based generative adversarial network for seismic data reconstruction and denoising. Li et al. [27] employed a Swin Transformer architecture based on self-attention mechanisms to suppress random seismic noise.

In deep learning-based seismic denoising, patch-based processing is widely adopted, driven by the high dimensionality of field seismic data and constraints on GPU memory and computational throughput. While this strategy effectively reduces the memory footprint and circumvents input-size limits for large-scale seismic records, it suffers from inherent drawbacks. Specifically, patch-wise processing breaks the global spatial continuity of seismic profiles: denoising performed independently on each patch lacks global contextual constraints, so the network captures only local features within the current window and cannot model the structural correlations of coherent seismic events across patches. When denoised patches are stitched back into a complete section, this commonly leads to amplitude inconsistencies and event discontinuities at patch boundaries. This problem is further exacerbated for seismic data from areas with complex geological structures. Beyond the patch strategy itself, convolutional neural network (CNN) architectures are intrinsically limited by their finite receptive fields, which hinders effective global modeling of high-resolution seismic data. Transformer-based methods offer stronger global modeling capacity, but their memory cost and computational complexity grow quadratically with sequence length, severely limiting their practical deployment for large-scale seismic denoising.

Several heuristic strategies, such as overlapping-window inference and weighted patch blending, have been used to mitigate boundary artifacts in patch-based denoising. However, these approaches primarily smooth transitions at patch edges and do not explicitly enforce the geometric consistency of coherent events across non-adjacent patches. For large land shot gathers, coherent reflections and structured noise can extend across multiple local windows, meaning that denoising decisions for a single patch should be governed by the global geometry of seismic events. This observation motivates the introduction of a global structural prior to constrain local patch denoising, rather than relying solely on post-processing boundary blending.

To address the above limitations and improve the global consistency of patch-based seismic denoising, we propose a denoising framework with fluctuation prior regularization, termed DFRSeisnet. The complete seismic data denoising process is explicitly decomposed into global denoising and local denoising. The signal variations and structural information of effective seismic signals obtained from the global branch are used to construct the fluctuation prior, which constrains the denoising of local patches. This strategy enhances the consistency between locally restored results and global seismic structures, mitigates stitching discontinuities induced by patch-wise processing. An integrated training framework is established to achieve globally consistent and robust denoising of seismic data under complex exploration environments. To overcome the respective limitations of CNN and Transformer architectures, we adopt the RMT block as the basic building unit. To further enhance the global consistency of local patch denoising, we introduce a local denoising strategy constrained by fluctuation priors, which allows local patches to incorporate implicit global information during denoising. In addition, to tackle the optimization imbalance between global and local tasks, we propose a grouped regularization strategy for the dual optimization objectives. This strategy integrates task-specific regularization terms, enabling the framework to capture the distinct characteristics of global and local objectives while dynamically balancing the corresponding task losses via an adaptive mechanism. Experiments on synthetic test datasets show that DFRSeisnet achieves superior denoising performance and yields improved global continuity in reconstructed full seismic sections. Validation on field seismic data, supported by velocity spectrum and stacking analysis, further demonstrates that the data processed by DFRSeisnet deliver high-quality denoised results that benefit subsequent seismic processing workflows.

The main contributions of this study are summarized as follows:

- We formulate large-section seismic background-noise attenuation as a globally constrained patch-wise denoising problem. A low-resolution global prediction is used to provide a fluctuation prior that guides local high-resolution denoising and mitigates stitching discontinuities across patch boundaries.
- We design a shared global–local denoising framework in which the global and local tasks are optimized in a common parameter space. This design allows the network to learn cross-scale representations of coherent seismic events while maintaining local detail recovery.
- We introduce a scale-uncertainty-aware weighting strategy to balance the global and local denoising objectives during training, and we quantify patch-stitching artifacts using a stitching jump ratio that reflects amplitude discontinuities along patch boundaries.
- We validate the method on synthetic noisy shot gathers generated from field signals and field-recorded background noise, and further evaluate its influence on field-data denoising, velocity-spectrum analysis, and stacked sections.

## 2. Materials and Methods

### 2.1. Framework Overview

The acquired seismic data can be modeled as the superposition of the primary seismic signal and background noise, following the additive noise formulation:

(1) y=s+n,

where *y* denotes the observed seismic record, *s* represents the source-associated primary (effective) signal, and *n* denotes the source-independent background noise.

For deep learning-based denoising, the network can be designed to predict either the effective signal directly or the noise component. Among the two paradigms, residual learning (i.e., predicting the noise term) generally yields better denoising performance [28], which can be formulated as:

(2) $$\hat{s}=y-m\left(y\right),$$

where m(y) denotes the noise predicted by the neural network from the observed seismic data *y*. The corresponding training objective is to minimize the discrepancy between the predicted noise and the ground-truth noise.

Driven by constraints on GPU memory and computational throughput, most deep learning-based seismic denoising workflows rely on a patch-based processing strategy: full seismic sections are partitioned into small local patches, each denoised independently. While this approach circumvents input-size limitations for large-scale seismic records, it disrupts the spatial continuity and structural coherence of subsurface reflection events. Because each patch is processed in isolation, the network cannot capture long-range structural correlations across patch boundaries. This commonly produces amplitude mismatches and event discontinuities at stitching locations, degrading the global consistency of the reconstructed denoised section. The underlying cause is the absence of explicit global structural constraints to guide local patch-level denoising.

To mitigate this limitation, we decompose seismic denoising into two coupled subtasks—a global optimization subtask and a local optimization subtask—and introduce a fluctuation prior extracted from the global denoising result to explicitly constrain local patch denoising, thereby improving the global structural consistency of the output. We formally define this fluctuation prior below.

Let D(·) denote a spatial downsampling operator, U(·) denote an upsampling operator that restores the original data resolution, and $C_{k}\left(\cdot \right)$ denote a cropping operator that extracts the spatial extent of the *k*-th local patch. For a full input seismic section *x*, we first generate its low-resolution counterpart $x_{d}=D\left(x\right)$, which is passed to a global denoising network ${\mathrm{Net}}_{G}$ parameterized by $\theta_{G}$ to yield a low-resolution global denoised estimate:

(3) $$x_{fp}={\mathrm{Net}}_{G}\left(x_{d};\theta_{G}\right).$$

Specifically, to facilitate network training and information interaction between global and local tasks, we set the block size processed by the global branch and the seismic patch size of the local branch equal to the network input size. Given the available computational resources, we carried out comparative experiments on different input sizes. Balancing computational cost and denoising performance, the network input size was finally set to 256 × 256. Subsequently, the full seismic data x is downsampled by a predefined downsampling factor to obtain $x_{d}$.

After upsampling $x_{fp}$ to the original resolution, we crop the result to align with the spatial bounds of the *k*-th local patch, yielding the fluctuation prior $p_{k}$ for that window:

(4) $$p_{k}=C_{k}\left[U\left({\mathrm{Net}}_{G}\left(D\left(x\right);\theta_{G}\right)\right)\right].$$

Specifically, the prior preserves the laterally continuous geometry of coherent reflection events and captures large-scale lateral fluctuations in event morphology and amplitude, rather than recovering high-resolution fine-scale features. Critically, this prior is not intended to replace the local noisy patch as input. Instead, it functions as a global structural constraint: it guides the local denoising branch to preserve consistency with the large-scale event trend during high-resolution local refinement, thereby suppressing boundary discontinuities that arise from independent patch processing.

**Figure 1:**
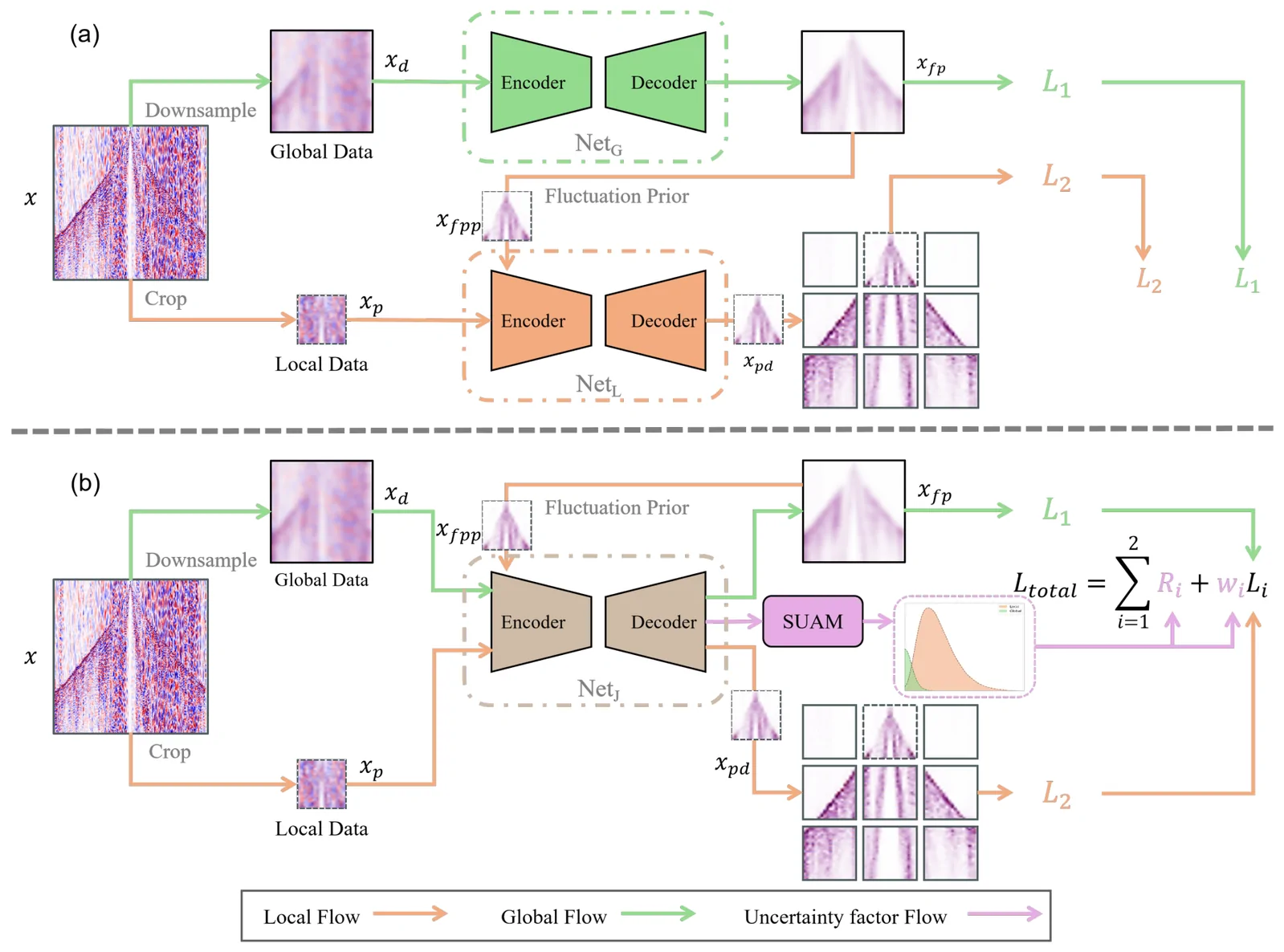
The overall framework: the complete seismic data denoising task is formulated as global and local optimization problems, where the fluctuation prior ensures the global consistency of local denoising. (**a**) A dual-task denoising framework with fluctuation priors, in which the global and local networks are independently optimized for their respective tasks. (**b**) The architecture of the proposed DFRSeisnet framework, where the scale-uncertainty-aware module adaptively optimizes the global and local tasks during the training process.

A naive implementation of this global–local denoising paradigm uses two separate networks under a multi-task learning framework: a global denoising network ${\mathrm{Net}}_{G}$ and a local denoising network ${\mathrm{Net}}_{L}$, as depicted in Figure 1a. In this architecture, the fluctuation prior $p_{k}$ is concatenated with the corresponding local noisy patch $x_{p}$ and fed into ${\mathrm{Net}}_{L}$, which is parameterized by $\theta_{L}$, to produce a locally refined denoised output $x_{pd}$:

(5) $$x_{pd}={\mathrm{Net}}_{L}\left(x_{p},p_{k};\theta_{L}\right).$$

The full denoised seismic section is then reconstructed by stitching all denoised local patches together.

This separate two-network design suffers from two key limitations. First, it inflates overall model complexity, and the fully decoupled parameter spaces prevent effective learning of shared cross-scale feature representations. Second, the two independent optimization streams lack explicit consistency regularization, meaning that errors accumulated in the global denoising stage can be amplified during local refinement, degrading both the stability and the global structural consistency of the final output.

Despite their difference in spatial scale, global and local denoising both target the same underlying structural patterns in seismic data, meaning that they can be modeled within a unified feature space. Motivated by this insight, we propose a fluctuation-prior-regularized denoising framework termed DFRSeisnet, shown in Figure 1b. In contrast to the dual-network architecture in Figure 1a, DFRSeisnet uses a single shared backbone network, denoted ${\mathrm{Net}}_{J}$, to jointly model both global and local denoising tasks. This unified design reduces the overall parameter complexity while enabling cross-scale feature sharing and collaborative optimization across the two subtasks.

Considering the spatial continuity and waveform variation characteristics of seismic data, bicubic interpolation provides smoother amplitude transitions during scaling transformation and is adopted as our scaling strategy. During training, the full noisy seismic section x is first downsampled via bicubic interpolation [29] to produce the low-resolution input $x_{d}$, which is passed through the shared network ${\mathrm{Net}}_{J}$ to generate the global denoised estimate.

(6) $$x_{fp}={\mathrm{Net}}_{J}\left(x_{d};\theta_{J}\right),$$

where $\theta_{J}$ denotes the learnable parameters of the shared backbone. Next, $x_{fp}$ is upsampled to the original resolution and cropped to match each local patch, yielding the global fluctuation prior $x_{fpp}$. This prior is concatenated with the corresponding original noisy local patch $x_{p}$ along the channel dimension and fed into the same shared network ${\mathrm{Net}}_{J}$ to produce the locally refined denoised output:

(7) $$x_{pd}={\mathrm{Net}}_{J}\left(x_{p},x_{fpp};\theta_{J}\right).$$

By sharing a single parameter space, DFRSeisnet learns both global structural consistency and local fine-grained texture features in a unified manner. It also explicitly uses structural information from the global denoising estimate to constrain the local restoration process, mitigating discontinuity artifacts at patch stitching boundaries. For supervised training, the global branch uses downsampled clean seismic sections as targets, while the local branch uses corresponding clean local patches as ground truth.

However, the two subtasks differ substantially in input data distribution and feature representation scale. A simple linear weighting of their respective loss functions cannot adaptively account for differences in optimization difficulty, often leading to imbalanced training convergence.

**Figure 2:**
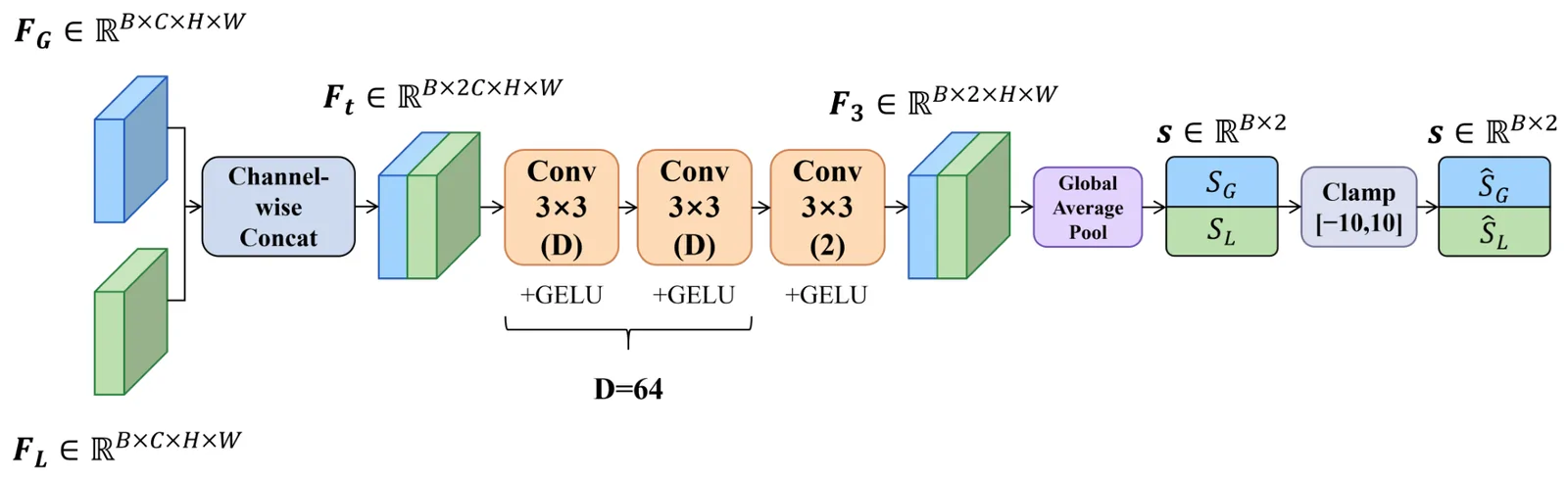
Structure diagram of the Scale-Aware Uncertainty Module. It consists of three layers of 3×3 convolutions, global average pooling, and a Clamp stabilization block.

To address the above issue, this paper introduces the Scale-Aware Uncertainty Module (SUAM), whose architecture is illustrated in Figure 2. It estimates task uncertainty separately from the deep features of the global and local tasks and converts the estimated uncertainty into corresponding loss weights, so as to realize adaptive dynamic balance between global and local tasks. Specifically, SUAM receives the bottleneck features output by the deepest RMT modules of the global and local branches, respectively. Let the bottleneck features of the global and local branches be denoted as $F_{G}\in R^{C\times H\times W}$ and $F_{L}\in R^{C\times H\times W}$, respectively. Since the two types of features have the same spatial dimensions, they are first concatenated along the channel dimension to obtain the joint feature:

(8) $$F_{t}=\mathrm{Concat}\left(F_{G},F_{L}\right)$$

The joint feature is then sequentially processed by three 3×3 convolutional layers for feature transformation. The first and second convolutional layers map the input channels to the hidden dimension *D*, which can be formulated as:

(9) $$F_{n}=\phi \left({\mathrm{Conv}}_{n}\left(F_{n-1}\right)\right),\;n\in \left\{1,2\right\}$$

where $F_{n}$ denotes the feature obtained after the *n*-th convolutional layer, ${\mathrm{Conv}}_{n}\left(\cdot \right)$ represents the *n*-th convolutional layer, and ϕ(·) denotes the GELU activation function. Both convolutional layers employ 3×3 kernels with a stride of 1 and a padding of 1, thereby progressively extracting joint feature representations while preserving the spatial resolution. The hidden dimension *D* is set to 64.

The third convolutional layer further projects the feature channels to 2, corresponding to the uncertainty representations of the global and local tasks, respectively. Global average pooling (GAP) is subsequently applied to obtain the log-variance parameters of the two tasks:

(10) $$s=\mathrm{GAP}\left({\mathrm{Conv}}_{3}\left(F_{2}\right)\right)\in R^{B\times 2}$$

Since the log-variance parameters are adaptively learned during training and are randomly initialized, excessively large or small uncertainty weights may lead to numerical instability during optimization. Therefore, explicit clamp is applied to the uncertainty parameters produced by SUAM:

(11) $$\hat{s}=\mathrm{clamp}\left(s,s_{\min},s_{\max}\right)$$

where the clipping range adopted in this work is [−10,10]. This range can effectively avoid numerical instability caused by extreme uncertainty estimation while retaining the adaptive adjustment capability of uncertainty.

Building on this module, we incorporate multi-task uncertainty regularization [30] into the overall optimization objective (detailed in Section 2.2). By adaptively estimating the uncertainty associated with each subtask, the framework dynamically adjusts the weight of each task loss, effectively mitigating optimization imbalance caused by differences in input distribution and feature scale. This design improves the network’s ability to jointly model global structural consistency and local fine-scale structural details.

### 2.2. Global and Local Uncertainty Regularization

In the proposed denoising framework shown in Figure 1b, the network performs denoising on the downsampled complete seismic data $x_{d}$ and on the local patch data $x_{p}$ combined with the resampled fluctuation prior $x_{fpp}$, respectively. Through the joint optimization of global and local tasks, the framework aims to obtain denoising results $x_{pd}$ that simultaneously preserve global consistency and local detail fidelity. However, during the joint optimization process, the two tasks exhibit significant differences in feature scales and statistical distributions. Specifically, the global task focuses on modeling low-frequency, large-scale structural information, whereas the local task emphasizes the representation of high-frequency, small-scale detail features. Such discrepancies lead to variance imbalance in cross-scale feature responses, which adversely affects the multitask optimization process. As a consequence, the network tends to preferentially fit one task during training, thereby suppressing the effective learning capability of the other task.

To address the aforementioned issue, we introduce a task uncertainty regularization mechanism into the global and local multitask learning framework. Specifically, a SUAM is constructed to perform data-driven parameterized modeling of task-related uncertainty by taking the deep feature representations of the global and local tasks as inputs. The task uncertainty is represented in the form of logarithmic variance. Under the maximum likelihood estimation framework [30], the logarithmic variance can be interpreted as a characterization of the observation noise level, and its estimated value is used to adaptively adjust the weights of different task loss terms, thereby dynamically balancing the gradient contributions of different tasks. Based on the logarithmic variances estimated by the SUAM for the global and local tasks, denoted as $s_{g}=\log\sigma_{g}^{2}$ and $s_{l}=\log\sigma_{l}^{2}$, respectively, the overall joint loss function is defined as follows:

(12) $$L_{\mathrm{total}}=\frac{1}{2}\exp\left(-s_{g}\right)L_{1}+\frac{1}{2}s_{g}+\frac{1}{2}\exp\left(-s_{l}\right)L_{2}+\frac{1}{2}s_{l},$$

where $s_{g}$ and $s_{l}$ denote the uncertainty parameters of the global and local tasks, respectively, which are adaptively learned by the SUAM. Correspondingly, exp(−s) is employed as the dynamic weight for the associated task loss term, enabling adaptive weighting during the optimization process. The regularization term $\frac{1}{2}s$ suppresses the unbounded growth of the uncertainty parameters, thereby ensuring the stability of the optimization process. The loss functions $L_{1}$ and $L_{2}$ correspond to the global and local tasks, respectively, both of which adopt the mean squared error (MSE) as the evaluation metric. Let $\hat{S}$ denote the denoised result and *S* denote the ground-truth seismic data; the MSE loss is defined as follows:

(13) $$L_{\mathrm{MSE}}=\frac{1}{MN}\sum_{i=1}^{M}\sum_{j=1}^{N}{\left(S_{i,j}-{\hat{S}}_{i,j}\right)}^{2}.$$

### 2.3. RMT-Based U-Shaped Network (RUNet)

**Figure 3:**
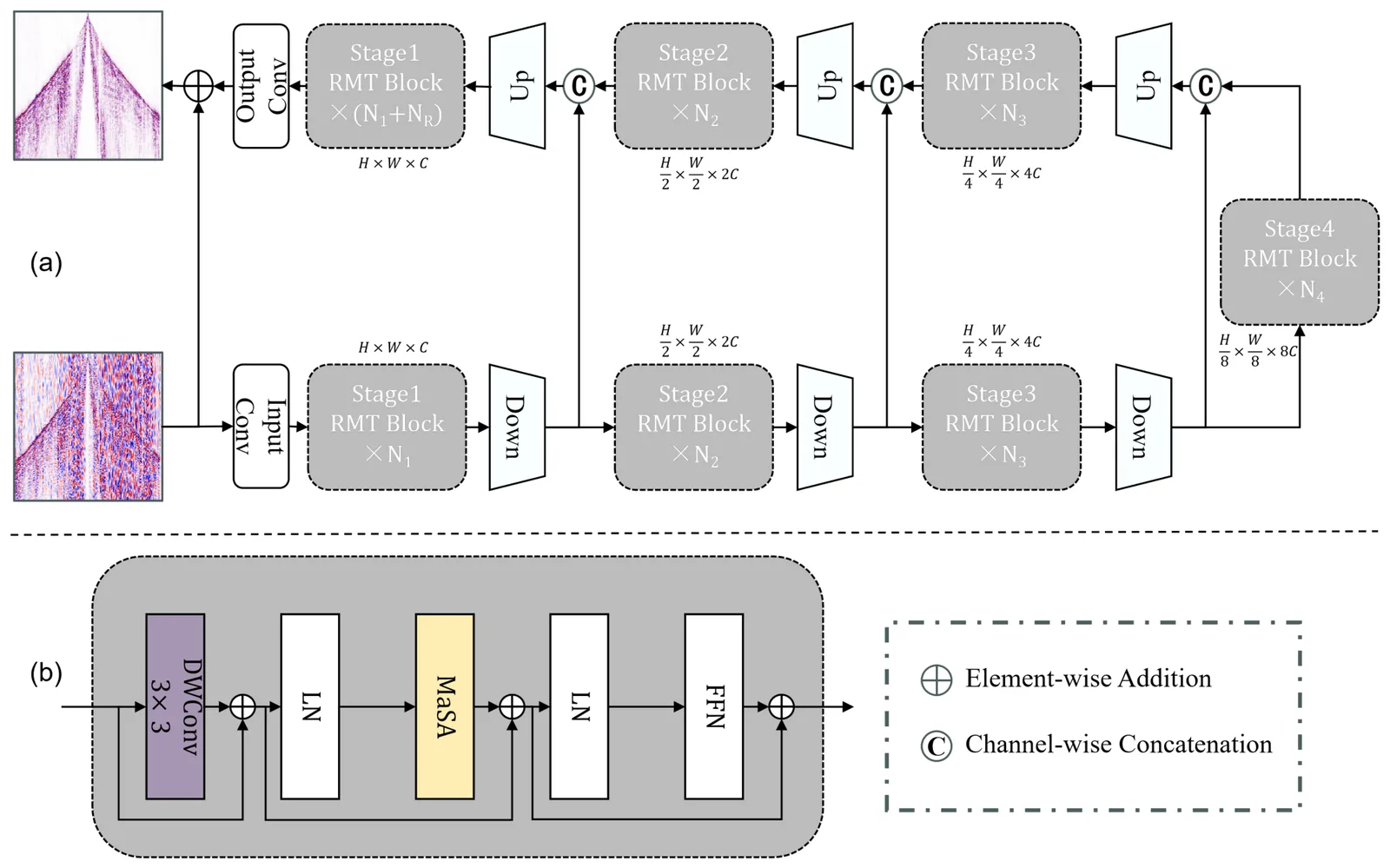
(**a**) Architecture of the denoising U-shaped network. (**b**) Illustration of the RMT block, which employs decomposable Manhattan self-attention to model global information with linear computational complexity.

We adopt the RMT [31] block as the basic feature extraction module to strengthen the global representation capability for high-resolution seismic data and reduce the computational cost of modeling long-range dependencies. By incorporating a Manhattan distance-based spatial decay function into the attention mechanism, the module introduces explicit spatial priors into the feature modeling process, enabling the network to effectively compensate for the limited receptive field of CNNs while capturing long-range dependencies [31]. Furthermore, RMT performs structured decomposition of the standard self-attention computation. This decomposition strategy effectively reduces the computational cost of global attention while preserving the Manhattan spatial decay prior and the global two-dimensional receptive field. As illustrated in Figure 3a, the RMT-based backbone adopts a four-stage U-shaped encoder–decoder architecture. Through three downsampling operations and the corresponding upsampling processes, the network performs multiscale feature extraction and reconstruction, thereby enhancing representation capability while maintaining computational efficiency [32].

In the early design RetNet adopted by RMT, for an input feature $X\in R^{N\times C}$, it first obtain the Query, Key, and Value features via linear projection:

(14) $$Q=\left(XW_{Q}\right)⊙\Theta ,\;K=\left(XW_{K}\right)⊙\bar{\Theta },\;V=XW_{V}$$

(15) $$\Theta_{n}=e^{in\theta },\;D_{nm}=\left\{\begin{array}{cc} \gamma^{n-m}, & n\geq m\\ 0, & n<m \end{array}\right.$$

(16) $$\mathrm{Retention}\left(X\right)=\left(QK^{⊤}⊙D\right)V$$

where $\bar{\Theta }$ denotes the complex conjugate of Θ, and D represents the two-dimensional decay matrix. In the image context, the two-dimensional spatial positions of the n-th and m-th tokens are denoted as $\left(x_{n},y_{n}\right)$ and $\left(x_{m},y_{m}\right)$, respectively. The two-dimensional spatial decay matrix is then defined as:

(17) $$D_{nm}^{2d}=\gamma^{\left|x_{n}-x_{m}\right|+\left|y_{n}-y_{m}\right|}$$

where γ∈(0,1) denotes the decay factor controlling the spatial decay rate, and $\left|x_{n}\;-\;x_{m}\right|+\left|y_{n}-y_{m}\right|$ represents the Manhattan distance between two tokens. As the spatial distance increases, the decay weight gradually decreases, thereby introducing explicit spatial priors into the global attention modeling process. Based on this spatial decay matrix, the Manhattan self-attention mechanism can be formulated as:

(18) $$\mathrm{MaSA}\left(X\right)=\left[\mathrm{Softmax}\left(QK^{⊤}\right)⊙D^{2d}\right]V$$

However, directly computing $QK^{⊤}$ requires constructing an N×N attention matrix, whose computational complexity grows quadratically with the number of tokens. For high-resolution feature maps in seismic data, a large *N* leads to prohibitive computational and memory overhead. To tackle this issue, RMT adopts a simple decomposition strategy. Specifically, RMT decomposes the self-attention computation and spatial decay matrix along horizontal and vertical directions, avoiding the direct construction of the full 2D attention matrix. Afterwards, the 1D bidirectional decay matrices are applied to these attention matrices.

(19) $$D_{nm}^{H}=\gamma^{\left|y_{n}-y_{m}\right|},\;D_{nm}^{W}=\gamma^{\left|x_{n}-x_{m}\right|}$$

(20) $${\mathrm{Attn}}_{H}=\mathrm{Softmax}\left(Q_{H}K_{H}^{⊤}\right)⊙D^{H}$$

(21) $${\mathrm{Attn}}_{W}=\mathrm{Softmax}\left(Q_{W}K_{W}^{⊤}\right)⊙D^{W}$$

where $D^{H}$ and $D^{W}$ denote the one-dimensional decay matrices along the two spatial directions. Finally, the decomposed Manhattan Self-Attention is obtained by sequentially aggregating the attention information from the two spatial directions:

(22) $${\mathrm{MaSA}}_{dec}\left(X\right)={\mathrm{Attn}}_{H}{\left({\mathrm{Attn}}_{W}V\right)}^{⊤}$$

From the perspective of computational complexity, suppose the size of the input feature map is H×W, then the number of tokens is N=HW. Standard global self-attention needs to compute $QK^{⊤}\in R^{N\times N}$, and its attention computational complexity is approximately $O(N^{2})$. In contrast, the decomposed MaSA computes one-dimensional attention along the two spatial directions separately, and its primary computational cost can be expressed as O((H+W)N).

For scale transformation, conventional pooling operations tend to cause irreversible loss of high-frequency seismic signal information. Therefore, during the downsampling and upsampling stages, a 1×1 convolution layer combined with pixel rearrangement operations is employed to achieve remapping between the spatial and channel dimensions [33]. This strategy not only improves computational efficiency but also facilitates the continuous propagation of structural information across different scales.

In addition, to alleviate the feature degradation problem that may arise in deep networks, symmetric skip connections are introduced to fuse the high-resolution features from the encoder stages with the high-level semantic representations from the decoder stages, thereby enhancing the completeness of feature representations. At the network output stage, the deep features generated by the decoder are mapped through a 3×3 convolution layer, followed by the introduction of a global residual learning strategy. Specifically, the input noisy data are added element-wise to the predicted output, enabling the network to focus on learning the noise component rather than the original signal itself. This design further promotes network convergence and stabilizes the training process to a certain extent [34].

## 3. Experiments

### 3.1. Evaluation Metrics

To comprehensively evaluate the quality of the denoising results and the severity of blocking artifacts, SNR and SSIM are employed as evaluation metrics, and a stitching discontinuity ratio is further designed to quantify the intensity of stitching artifacts. SNR is employed to evaluate the quality of the denoising results, and its calculation is defined as follows:

(23) $$\mathrm{SNR}=10\log_{10}\left(\frac{∥x{∥}_{2}^{2}}{∥x-\hat{x}{∥}_{2}^{2}}\right),$$

where *x* denotes the clean seismic data sample, $\hat{x}$ represents the predicted result, and $∥\cdot {∥}_{2}^{2}$ denotes the squared $L_{2}$-norm. A higher SNR value indicates better denoising performance and more effective background noise suppression. SSIM is employed to evaluate structural similarity and is defined as follows:

(24) $$\mathrm{SSIM}\left(s,\hat{s}\right)=\frac{\left(2\mu_{s}\mu_{\hat{s}}+C_{1}\right)\left(2\sigma_{s\hat{s}}+C_{2}\right)}{\left(\mu_{s}^{2}+\mu_{\hat{s}}^{2}+C_{1}\right)\left(\sigma_{s}^{2}+\sigma_{\hat{s}}^{2}+C_{2}\right)},$$

where μ denotes the mean, $\sigma^{2}$ represents the variance, and $\sigma_{s\hat{s}}$ denotes the covariance. $C_{1}$ and $C_{2}$ are stability constants. However, these metrics primarily reflect the overall error and structural similarity between the predicted and ground-truth results, making them insufficient for characterizing the local stitching discontinuities introduced by patch-wise processing. To address this issue, considering that seismic data exhibit strong coherent event continuity and that adjacent traces and samples should maintain high correlations [35], we propose a quantitative metric termed the Stitching Jump Ratio (SJR) to evaluate stitching discontinuities. Specifically, SJR quantitatively assesses the continuity of stitching regions by comparing the amplitude differences between adjacent data points at stitching boundaries with the global average amplitude difference. The metric is defined as follows:

(25) $$\mathrm{SJR}=0.5\left({\mathrm{SJR}}_{t}+{\mathrm{SJR}}_{x}\right),$$

where ${\mathrm{SJR}}_{t}$ measures amplitude discontinuities along the temporal (depth) direction at horizontal stitching boundaries, and ${\mathrm{SJR}}_{x}$ evaluates amplitude jumps along the cross-trace (spatial) direction at vertical stitching boundaries. The two components are formulated as:

(26) $${\mathrm{SJR}}_{t}=\frac{\frac{1}{|\Omega_{b}^{t}|}\sum_{r\in \Omega_{b}^{t}}\frac{1}{W}\sum_{j=1}^{W}\left|S(r,j)-S(r-1,j)\right|}{\frac{1}{(H-1)W}\sum_{i=1}^{H-1}\sum_{j=1}^{W}\left|S(i+1,j)-S(i,j)\right|},$$

(27) $${\mathrm{SJR}}_{x}=\frac{\frac{1}{|\Omega_{b}^{x}|}\sum_{c\in \Omega_{b}^{x}}\frac{1}{H}\sum_{i=1}^{H}\left|S(i,c)-S(i,c-1)\right|}{\frac{1}{H(W-1)}\sum_{i=1}^{H}\sum_{j=1}^{W-1}\left|S(i,j+1)-S(i,j)\right|},$$

Here, $\Omega_{b}^{t}$ denotes the set of row indices corresponding to temporal-direction stitching boundaries, and $\Omega_{b}^{x}$ denotes the set of column indices corresponding to cross-trace stitching boundaries. *H* and *W* represent the number of temporal sampling points and seismic traces in the seismic section, respectively, and S(i,j) denotes the amplitude value at the *i*-th sampling point and *j*-th trace.

SJR quantifies the ratio of amplitude discontinuities at patch stitching boundaries to the natural amplitude variations of the entire seismic section. A value close to 1 indicates negligible stitching artifacts, while a value significantly greater than 1 indicates severe amplitude discontinuities and structural distortion at stitching positions.

### 3.2. Dataset

In practical field seismic acquisition, strictly paired noisy and noise-free seismic records are generally unavailable for fully supervised denoising model training. To address this limitation, we construct training data pairs using field seismic data from the same survey area: relatively high SNR shot gathers are adopted as approximate references for effective reflection signals, and noise records acquired under source-free conditions are used to represent realistic ambient background noise.

Given the inherent variability in the amplitude ratio between effective reflection signals and background noise for seismic data acquired under complex field acquisition conditions, we construct training samples covering a range of noise levels to enhance the model’s generalization robustness. Specifically, field-collected background noise is injected into clean reference gathers to achieve target signal-to-noise ratio (SNR) levels spanning from −5 dB to 5 dB. Derived analytically from the standard decibel SNR formulation, the noise injection process is expressed as:

(28) $$y=s+n\sqrt{\frac{\sum s^{2}}{10^{\mathrm{SNR}/10}\cdot \sum n^{2}}},$$

where *s* denotes the clean reference seismic signal, *n* represents the field background noise record, and both summations are computed over all sampling points of the seismic trace. The term $10^{\mathrm{SNR}/10}$ follows the standard power ratio conversion rule for decibel-scale SNR. Subsequently, the data are normalized to construct the final training sample pairs, and the normalization process is defined as follows:

(29) $$y_{\mathrm{norm}}=2\cdot \frac{y-y_{\min}}{y_{\max}-y_{\min}}-1,$$

where $y_{\min}$ and $y_{\max}$ denote the minimum and maximum values of the data, respectively. The experimental data were obtained from two real seismic acquisition datasets collected in northwestern China. Specifically, Dataset-L has a sampling interval of 4 ms and contains 521 relatively clean complete seismic records, each with a size of 1000×480. Dataset-S has a sampling interval of 2 ms and also contains 521 relatively clean complete seismic records, with each shot gather having a size of 2000×500. Given the discrepancies in sampling intervals and data dimensions of the two datasets, we perform separate training and testing for each dataset, and no joint training is adopted. For each dataset, 10,000 paired training samples were generated and divided into training, validation, and testing sets according to a ratio of 8:1:1. To avoid leakage between training and testing, the split was performed at the complete-shot-gather level before patch extraction and noise mixing. No local patches derived from the same original gather were shared across the training, validation, and test subsets.

### 3.3. Training Configuration

During training, the numbers of RMT blocks $N_{i}$ at different stages were set to [4,6,6,8], while $N_{R}$ was set to 4. The number of hidden channels *C* of RUNet was set to 48. The network input size was configured as 256×256, and the batch size was set to 16. The AdamW optimizer was adopted with an initial learning rate of $1\times 10^{-4}$. The network was trained for 100 epochs on a single NVIDIA RTX 4090 GPU.

Three representative methods were selected for comparative experiments, namely Wavelet, MSSA-Net [24], and SwinT [27]. Wavelet denotes the conventional denoising method. In the comparative experiments, the Symlet 8 wavelet basis was adopted with 4-level wavelet decomposition. MSSA-Net represents convolution- and fully connected-based methods, and SwinT represents Transformer-based attention methods, employing a Swin Transformer encoder–decoder architecture for network design. To ensure a fair comparison, all learning-based methods were trained using the same training, validation, and test splits and the same hardware environment. For the conventional wavelet method, parameters were selected on the validation set and then fixed for all test cases.

### 3.4. Test Set Comparison Experiment

**Figure 4:**
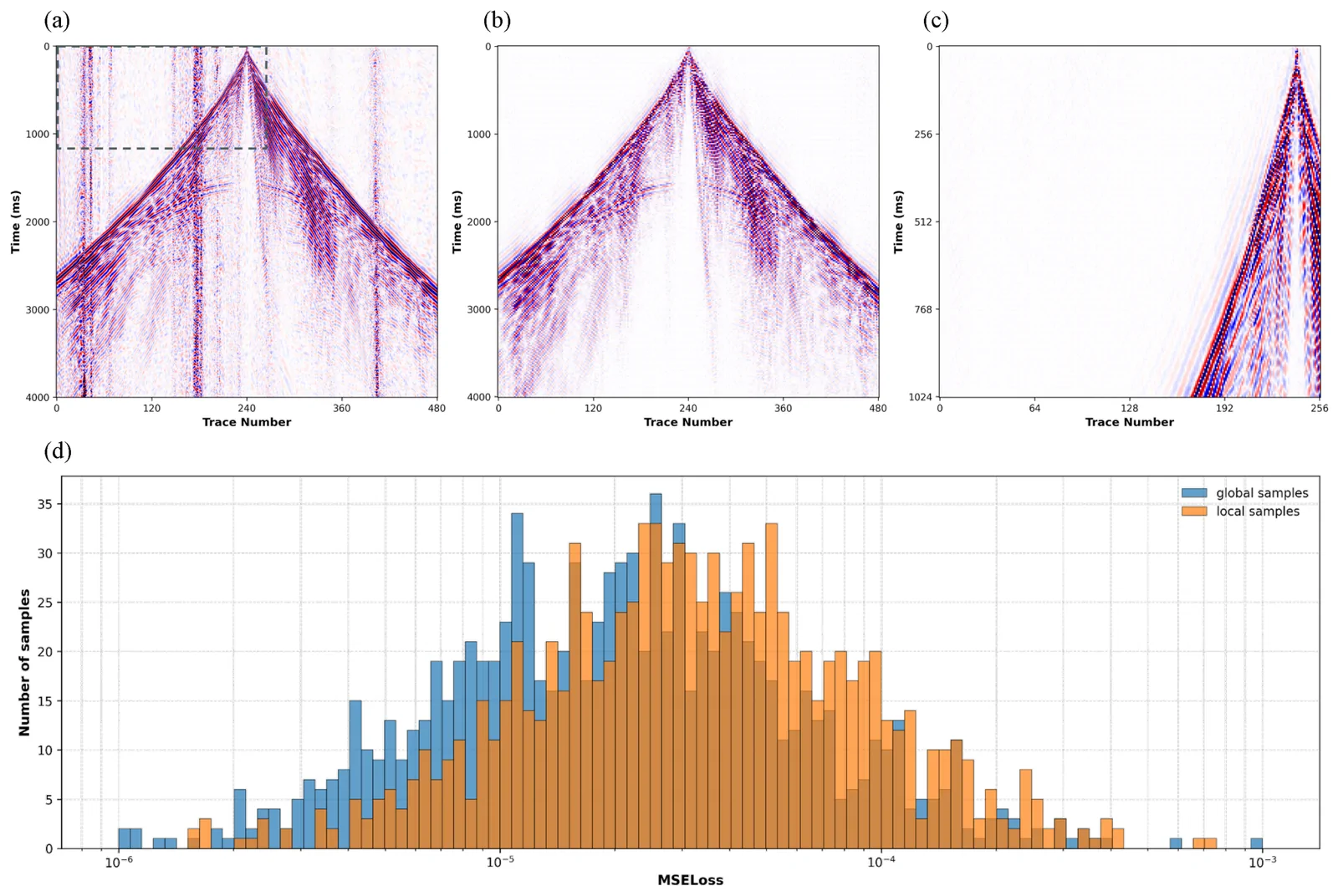
Global and local denoising results of complete noisy seismic data. (**a**) Complete noisy seismic data. (**b**) Global denoising result of the downsampled complete seismic data. (**c**) Local denoising result of the patch region marked by the dashed box in (**a**). (**d**) MSE statistics between the global and local denoising results and the corresponding clean ground-truth labels on the test set.

To verify that the network can correctly perform both global and local denoising—namely, accurately denoise the downsampled complete seismic data and effectively use the fluctuation priors generated by global denoising for local denoising—we conducted global and local denoising experiments on complete noisy seismic data, as illustrated in Figure 4. Specifically, the complete seismic data shown in Figure 4a are first downsampled and then fed into the network to generate the global denoising result shown in Figure 4b. Subsequently, the fluctuation prior extracted through resampling is combined with the local noisy patches and processed by the network to produce locally denoised results with global consistency, as shown in Figure 4c. In addition, the MSE histograms between the global denoising results, local denoising results, and their corresponding ground-truth labels are statistically analyzed, as illustrated in Figure 4d. The network exhibits stable denoising capability for both global and local tasks. There is a notable difference in optimization difficulty between the two tasks: the MSE values of the local denoising results are mainly concentrated around $4\times 10^{-5}$, whereas those of the global denoising results are primarily concentrated around $2\times 10^{-5}$. These results demonstrate that the proposed network can accurately perform both global and local denoising tasks.

**Figure 5:**
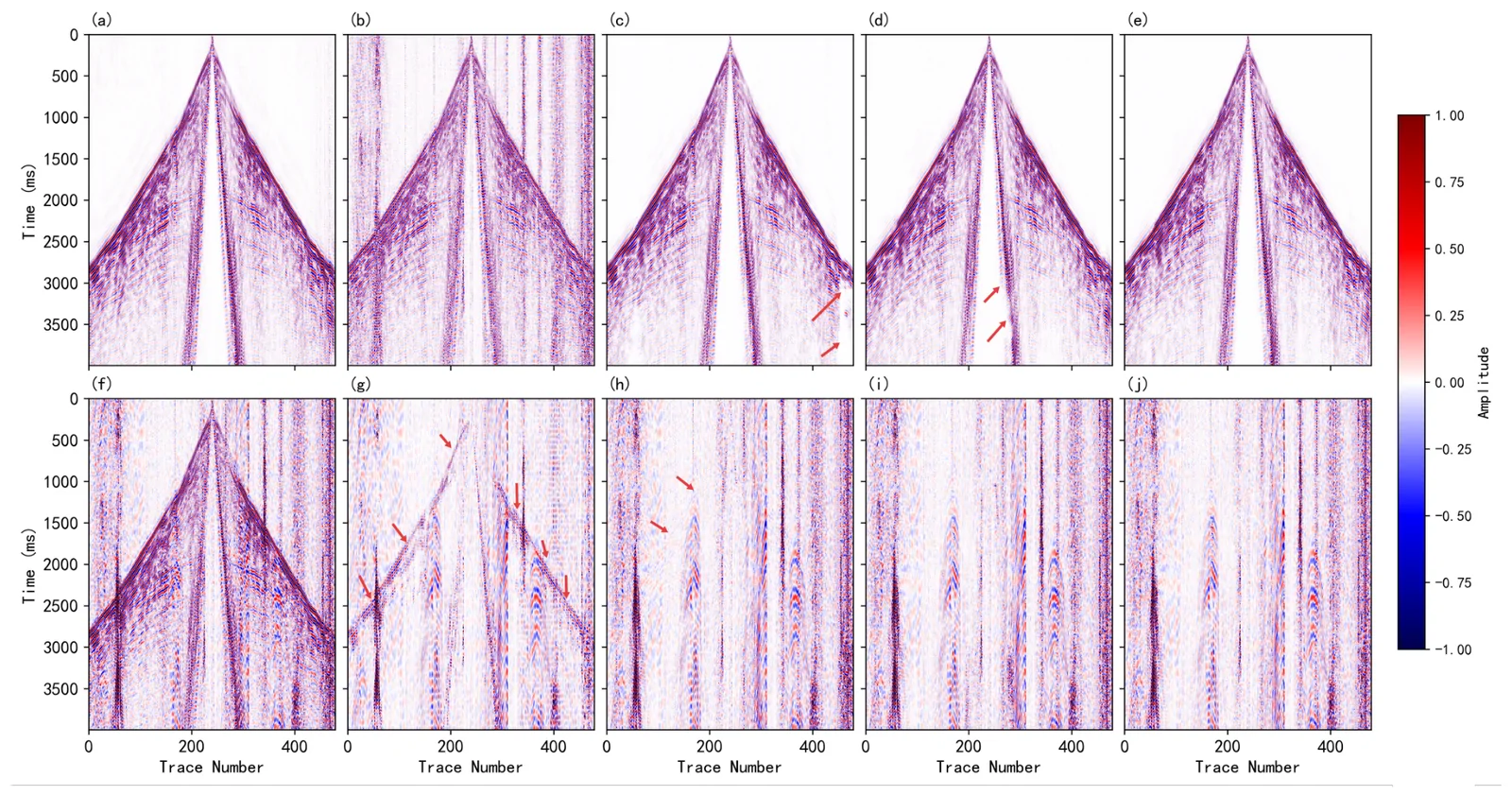
Comparison of denoising results on the test set with SNR = −5 dB. (**a**) Clean data. (**b**–**e**) Denoising results obtained by DFRSeisnet, SwinT, MSSA-Net, and Wavelet methods, respectively. (**f**) Noisy data. (**g**–**j**) Background noise components removed by DFRSeisnet, SwinT, MSSA-Net, and Wavelet methods, respectively.

To evaluate the effectiveness of the proposed method, noisy seismic data with an SNR of −5 dB from the test set were used for denoising experiments, and the denoising results together with the removed noise components are illustrated in Figure 5. The proposed method effectively suppresses background noise while preserving global consistency, which is visually reflected in the overall continuity of the stitched denoising result shown in Figure 5b. In contrast, the conventional patch-based CNN method MSSA-Net, shown in Figure 5d, and the Transformer-based method SwinT, shown in Figure 5c, both process seismic data on individual patches independently. As a result, the lack of global structural constraints leads to obvious stitching artifacts in their overall denoising results. Although the Wavelet-based method shown in Figure 5e processes the complete seismic data directly and therefore does not suffer from stitching artifacts, its denoising performance is relatively poor and causes noticeable damage to the effective seismic signals.

**Figure 6:**
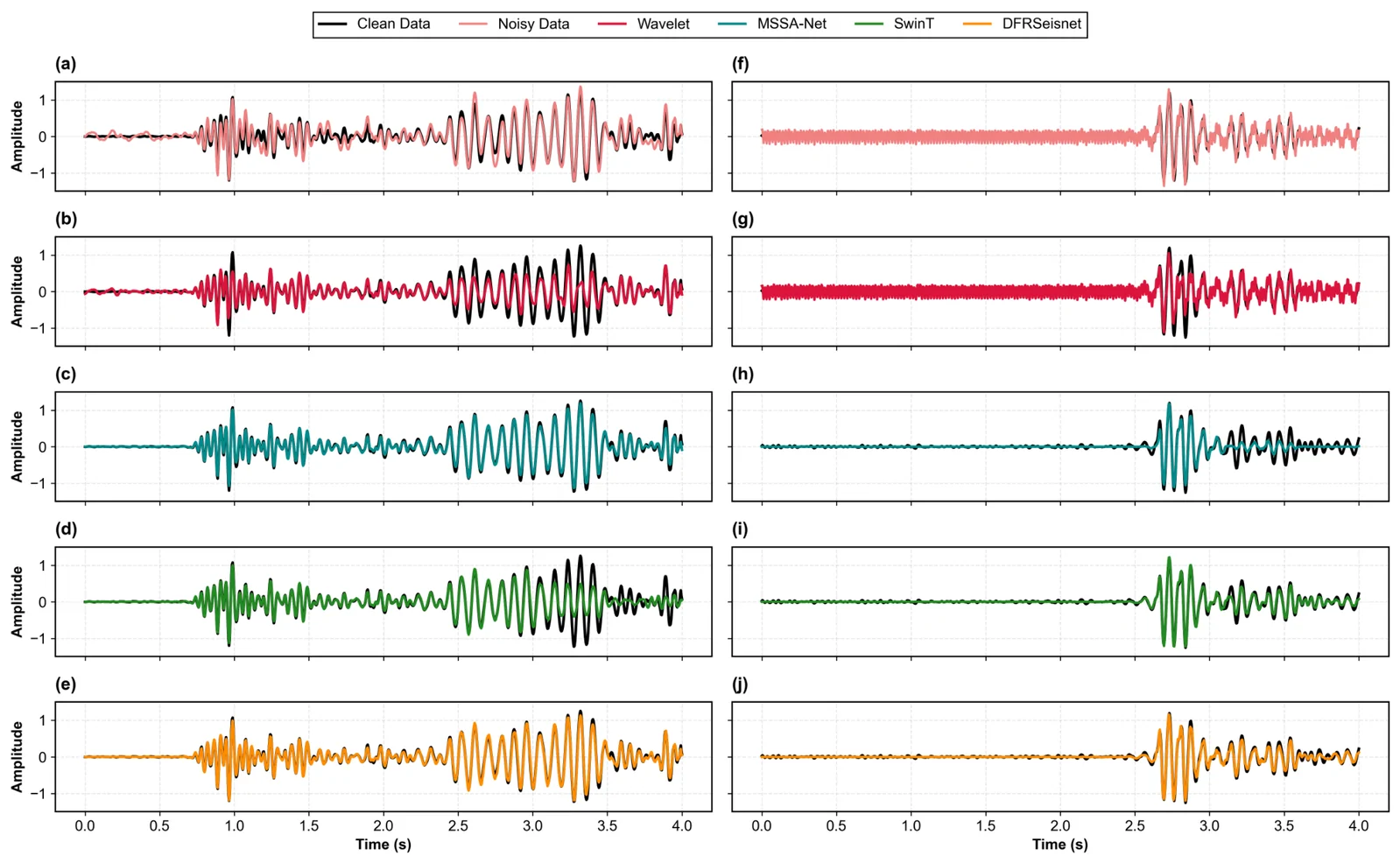
Single-trace analysis of the denoising results obtained by different methods in Figure 5. (**a**–**e**) Waveform comparisons at Trace 282 for noisy data and different denoising methods. (**f**–**j**) Waveform comparisons at Trace 451 for noisy data and different denoising methods.

**Figure 7:**
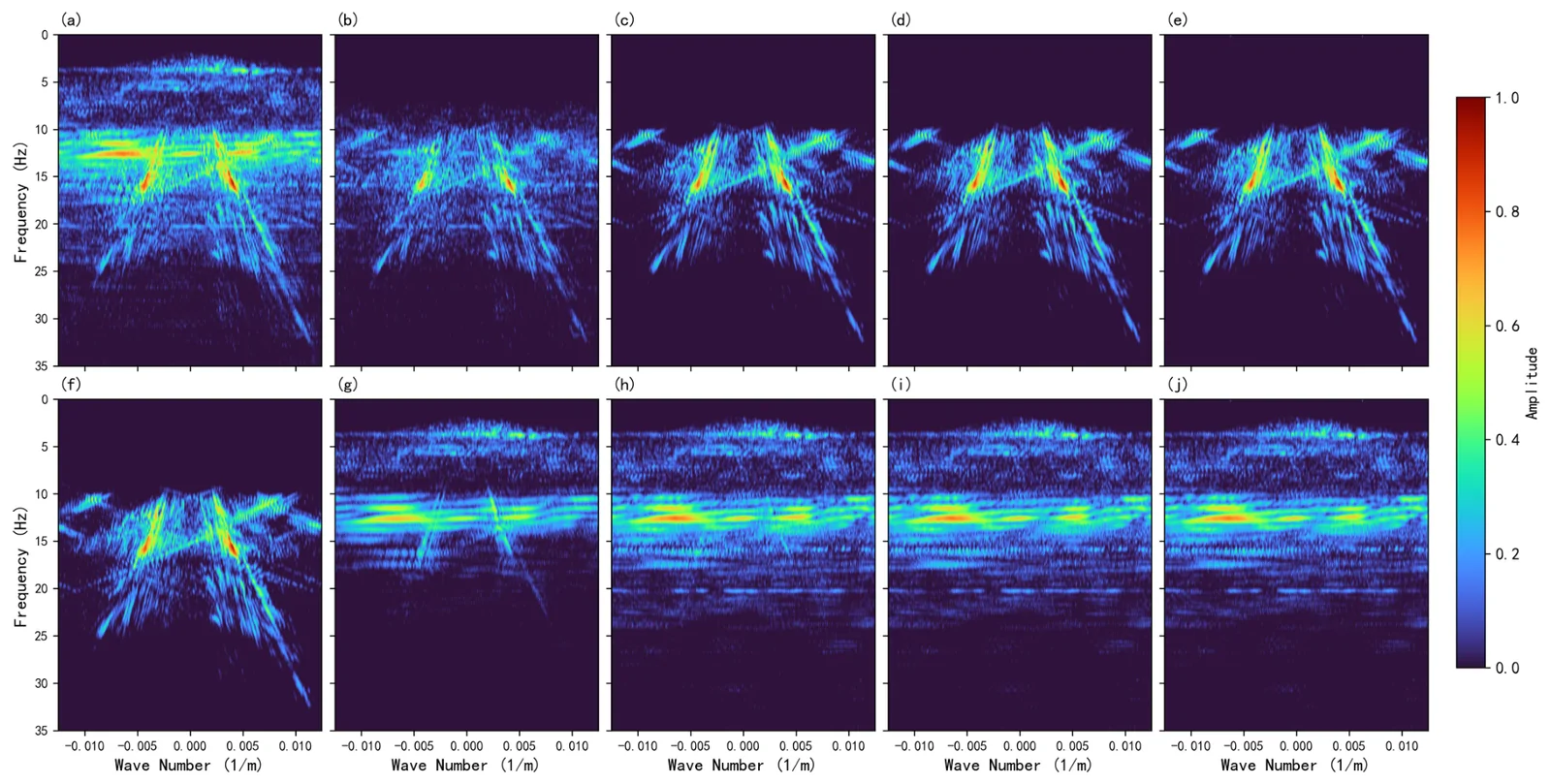
Frequency–wavenumber spectra of the denoising results and removed components for the test example in Figure 5. (**a**) Noisy data. (**b**–**e**) Spectra of the denoised results obtained by Wavelet, MSSA-Net, SwinT, and DFRSeisnet, respectively. (**f**) Clean data. (**g**–**j**) Spectra of the removed components obtained by Wavelet, MSSA-Net, SwinT, and DFRSeisnet, respectively.

We further analyzed the denoising results in both the time domain and the frequency–wavenumber (*f*–*k*) domain. Specifically, the waveforms of Trace 282 and Trace 451 from the denoising results of different methods shown in Figure 5 were analyzed, as illustrated in Figure 6. The results demonstrate that, compared with the other three methods, DFRSeisnet exhibits superior signal preservation capability. Among the four methods, the seismic signals denoised by DFRSeisnet are the closest to the clean ground-truth signals. As shown in Figure 6d,h, which correspond to the denoising results of SwinT on Trace 282 and MSSA-Net on Trace 451, respectively, both methods exhibit obvious energy attenuation after 3072 ms. This phenomenon corresponds to the blocking artifacts observed in Figure 5 and provides a direct manifestation of stitching discontinuities in the single-trace waveforms. The *f*–*k* spectra further indicate that DFRSeisnet outperforms the other deep learning methods in suppressing most background noise while preserving effective seismic signals. As illustrated in Figure 7, although the denoising results of all four methods exhibit generally consistent distributions within the dominant frequency bands of the effective signals, the *f*–*k* spectra of the residual noise components, obtained from the difference between the noisy data and denoised results, reveal significant effective signal leakage in the residual components of both the Wavelet and MSSA-Net methods. In addition, the *f*–*k* spectrum of the Wavelet-denoised result still contains a considerable amount of residual random noise. Although no obvious effective signal leakage is observed in the residual component of SwinT, its signal preservation capability in the time-domain waveforms remains inferior to that of the proposed DFRSeisnet.

Although the training samples were generated within the SNR range of −5 to 5 dB, we additionally evaluated the trained model at −10 and 10 dB to test its extrapolation ability under stronger and weaker noise conditions. The detailed results are presented in Table 1. The experimental results demonstrate that the proposed method achieves the best performance across all three core metrics, namely SNR, SSIM, and SJR. These results indicate that the proposed method improves both denoising quality and global consistency for the tested synthetic noisy datasets. In contrast, the Wavelet-based denoising method achieves the poorest performance. This limitation mainly arises from the idealized theoretical assumption that noise and effective signals are separable, as well as the inherent shortcomings associated with empirical parameter selection. The patch-based deep learning methods, MSSA-Net and SwinT, can effectively improve the SNR and structural similarity of the denoised results. However, under low-SNR conditions, the stitching jump ratio rises markedly, indicating the presence of obvious energy discontinuities at patch stitching boundaries. By comparison, the proposed method maintains a relatively stable stitching jump ratio under both low- and high-SNR denoising scenarios.

**Figure 8:**
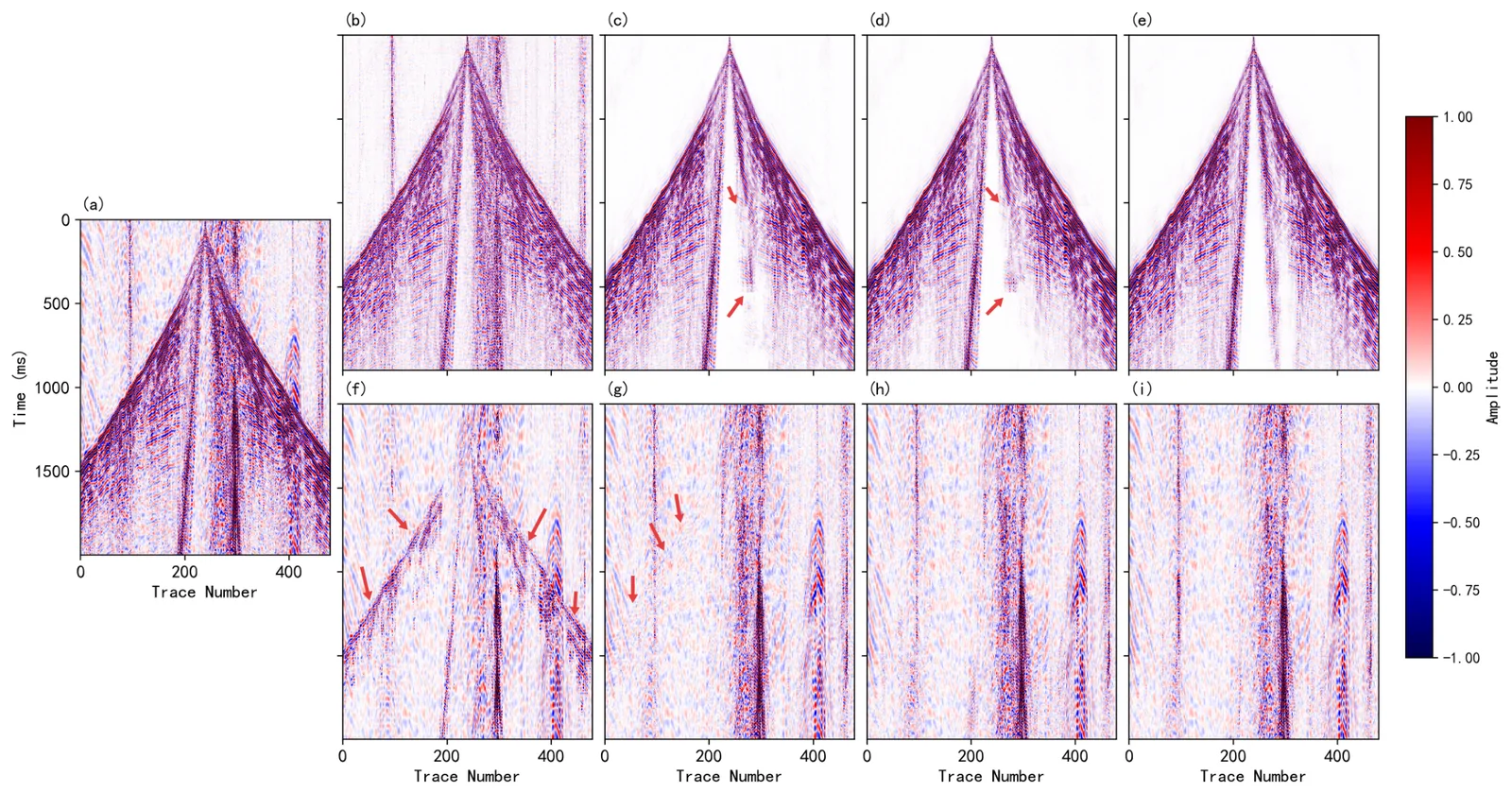
Denoising results on Dataset-L field noisy data: (**a**) noisy data; (**b**–**e**) denoising results obtained by Wavelet, MSSA-Net, SwinT, and DFRSeisnet, respectively; (**f**–**i**) noise components removed by Wavelet, MSSA-Net, SwinT, and DFRSeisnet, respectively. The marked arrows indicate the performance differences between different Methods.

**Figure 9:**
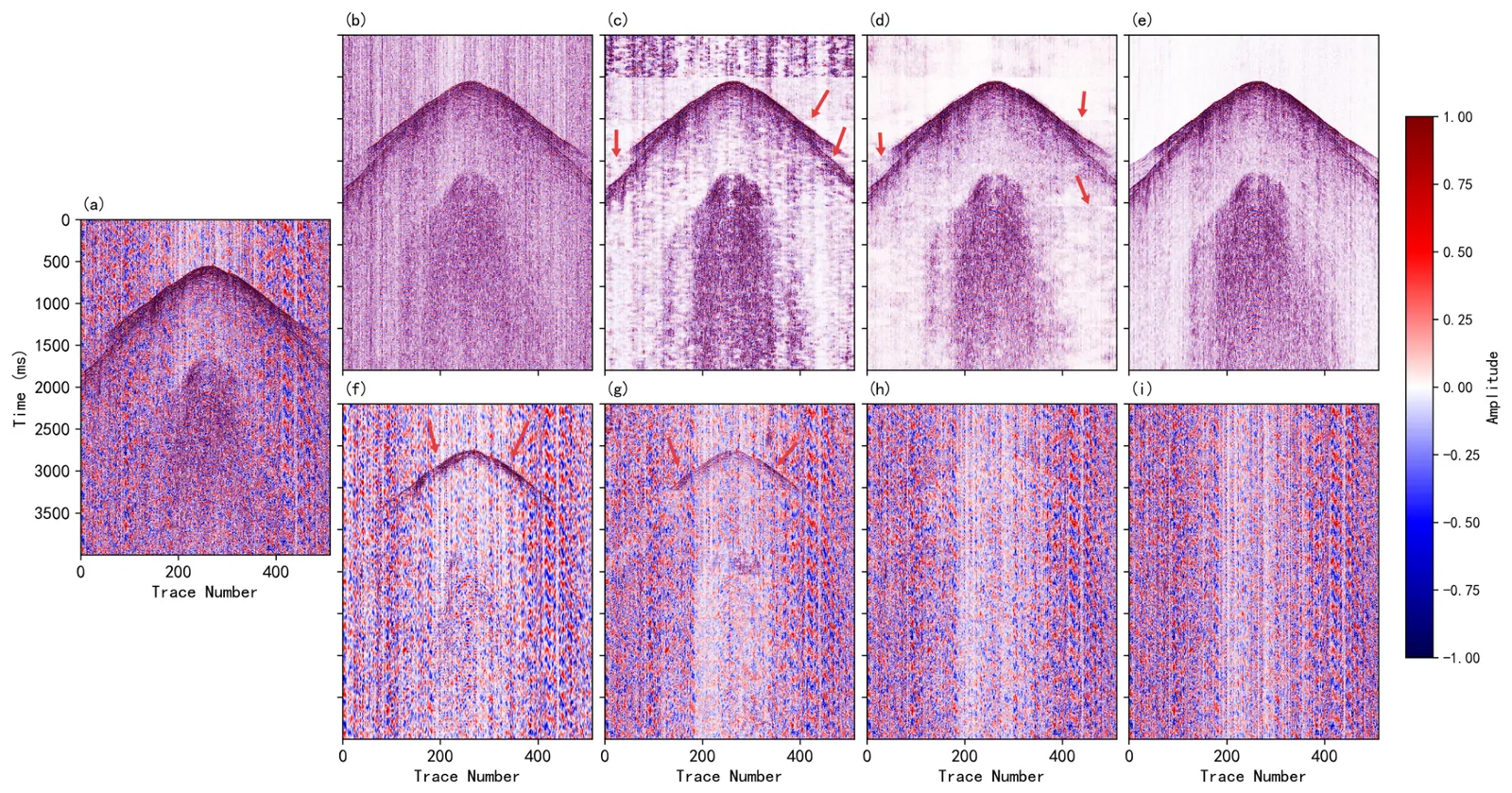
Denoising results on Dataset-S field noisy data: (**a**) noisy data; (**b**–**e**) denoising results obtained by Wavelet, MSSA-Net, SwinT, and DFRSeisnet, respectively; (**f**–**i**) noise components removed by Wavelet, MSSA-Net, SwinT, and DFRSeisnet, respectively. The marked arrows indicate the performance differences between different Methods.

To evaluate the denoising performance of the network on real low-SNR field data, we conducted denoising experiments on seismic records acquired from the same survey area that are heavily contaminated by strong background noise. The network trained on the artificially mixed-noise Dataset-L and Dataset-S was directly applied to denoise the field-acquired data affected by background interference. Figure 8 and Figure 9 present the denoising results of real low-SNR seismic data from Dataset-L and Dataset-S, respectively. The Wavelet-based method visibly reduces part of the background energy, but substantial residual noise remains. In contrast, MSSA-Net and SwinT effectively remove much of the noise. However, due to their patch-wise processing strategy, inconsistencies arise between adjacent patches, leading to evident blocking artifacts, as indicated by the red arrows in Figure 8 and Figure 9.

### 3.5. Ablation Study

**Figure 10:**
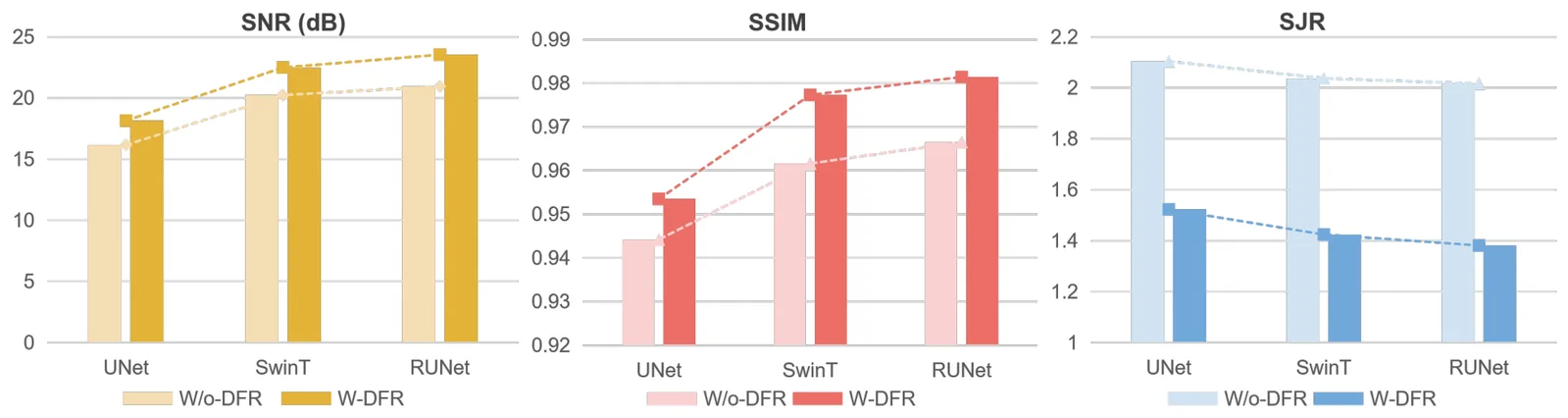
Ablation study: average values of three evaluation metrics on the validation set when the encoder–decoder adopts UNet, SwinT, or RUNet, with and without the proposed framework.

To verify the adaptability of the proposed framework across different types of encoder–decoder architectures, we conducted ablation experiments. Specifically, we replaced the encoder–decoder in the denoising framework with UNet, SwinT, and RUNet, respectively, and trained and tested these configurations on the same dataset. The average performance of three evaluation metrics on the validation set was then computed. The results are shown in Figure 10. Compared with the single patch-based denoising strategy, all types of encoder–decoder architectures achieve improved denoising performance when integrated into the proposed framework, while the stitching artifacts are also effectively alleviated. The best performance is achieved when RUNet is incorporated into the proposed framework. Table 2 presents the detailed average values of SNR, SSIM, and SJR on the validation set for UNet, SwinT, and RUNet with and without the proposed framework. The bolded values indicate the best results.

## 4. Results and Discussion

### 4.1. Impact of Denoising on Subsequent Processing

**Figure 11:**
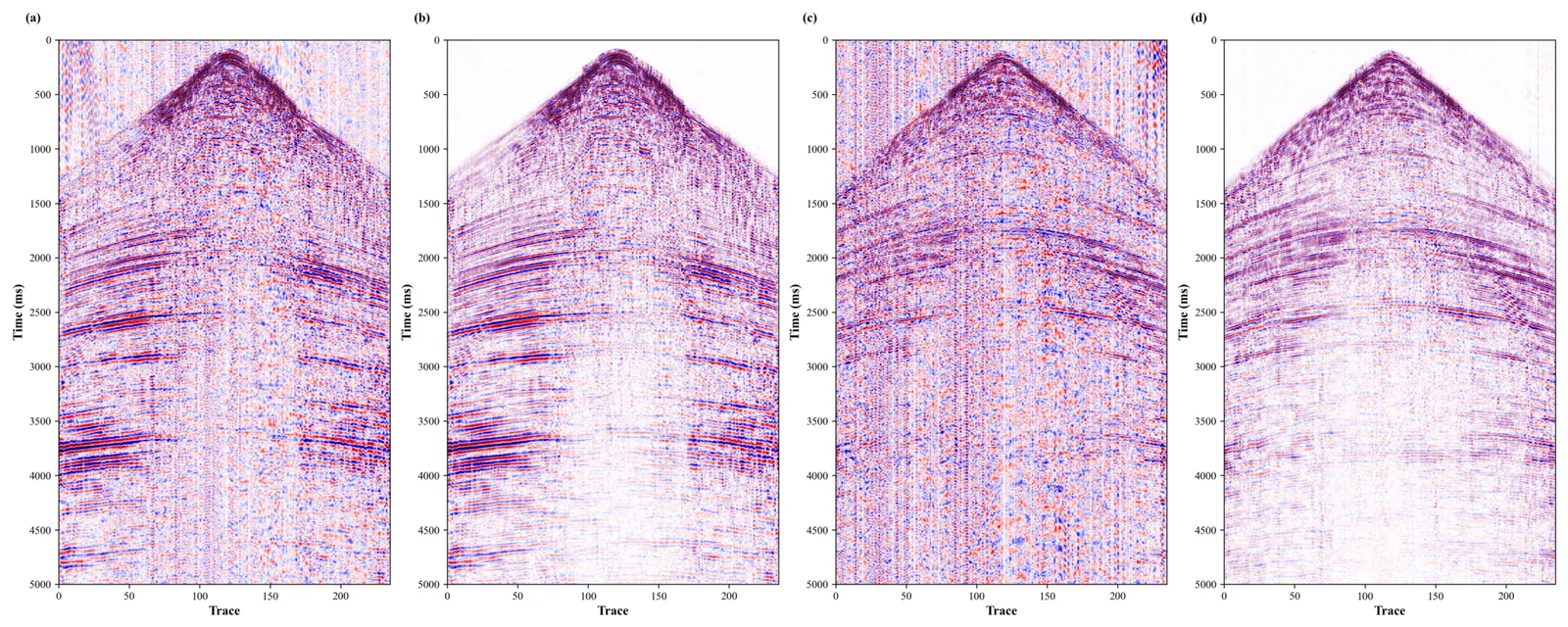
Denoising results of CSP gathers: (**a**) original data of field record No. 0500243; (**b**) denoised result of field record No. 0500243; (**c**) original data of field record No. 0500222; (**d**) denoised result of field record No. 0500222.

To evaluate the impact of background noise removal on subsequent seismic processing steps, we used field seismic data acquired from southwestern China. The data have a sampling interval of 2 ms and contain a total of 1,099,779 traces, with each trace consisting of 2500 sampling points. Based on relatively clean data from the same survey area and background noise recorded within the same region, we constructed Dataset-V for further validation. The network was trained using the same training strategy, and denoising was performed on Dataset-V in the form of common-shot-point (CSP) gathers. Figure 11 presents the denoising results of a subset of the data, demonstrating that most of the background noise in the CSP gathers has been effectively removed.

**Figure 12:**
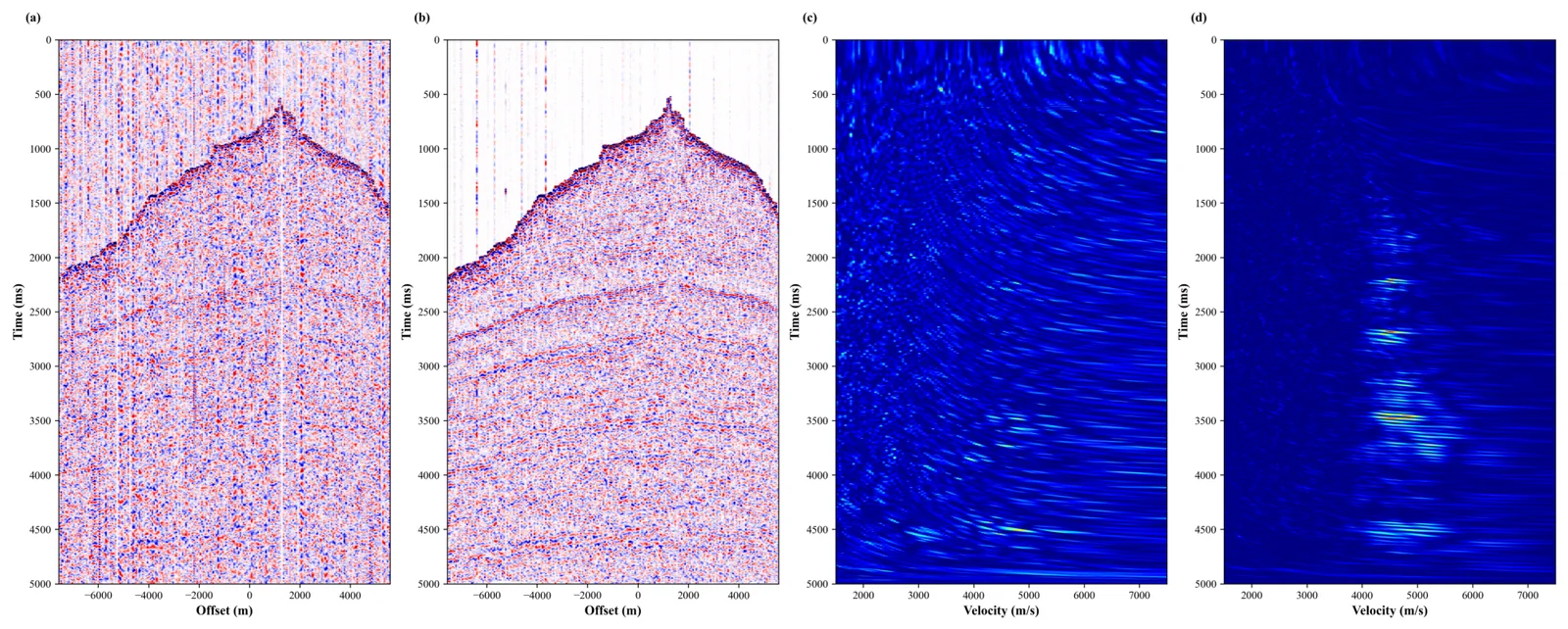
CMP velocity analysis extracted from CSP at line No. 721, point No. 1254: (**a**) CMP extracted from the original data; (**b**) CMP extracted from the denoised data; (**c**) velocity spectrum generated from the CMP extracted from the original data; (**d**) velocity spectrum generated from the CMP extracted from the denoised data.

To investigate the impact of denoising on velocity spectrum construction, common-mid-point (CMP) gathers from line No. 721 (trace range 974–3373) were extracted from CSP gathers before and after denoising. CMP gathers with fold greater than 80 were selected for velocity spectrum analysis, with a velocity scanning range of 1500–7500 m/s and a step size of 50 m/s. Figure 12 presents a comparison of the CMP gather (line No. 721, trace No. 1254, fold = 207) and its corresponding velocity spectra extracted before and after CSP denoising. Specifically, Figure 12a,c show the CMP gather and its velocity spectrum extracted from the original CSP, respectively, while Figure 12b,d show those extracted from the denoised CSP. After background noise suppression, the energy in the velocity spectrum becomes more concentrated within a reasonable effective velocity range, leading to more focused velocity features. In contrast, the velocity spectrum derived from the original noisy data exhibits a more dispersed energy distribution over a wider velocity range, making it difficult to accurately identify the effective velocity interval.

**Figure 13:**
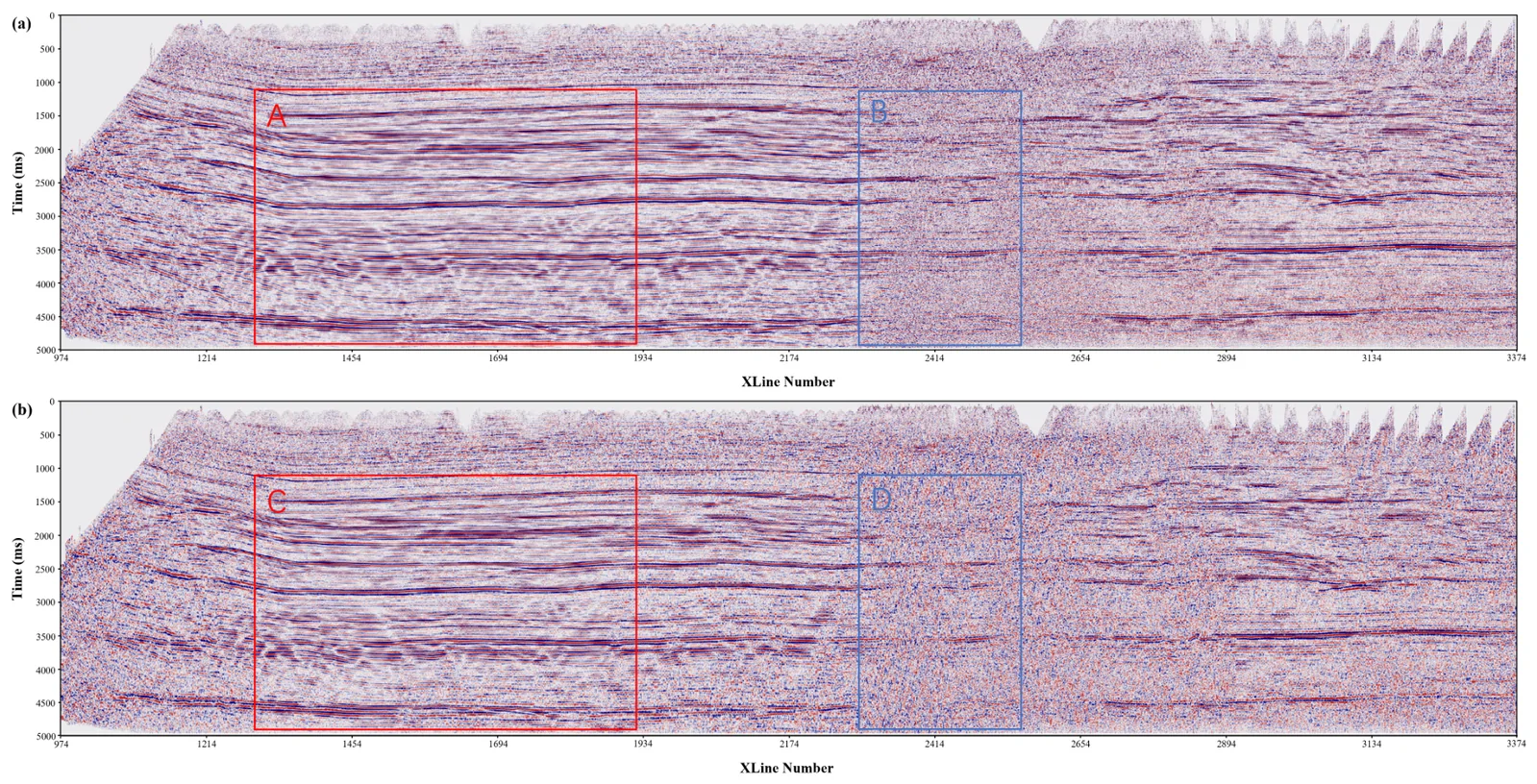
Comparison of stacked sections: (**a**) stacked section after denoising; (**b**) stacked section of the original data. Boxes A and C indicate the high-fold region in the denoised and original stacked sections, respectively; boxes B and D indicate the low-fold region.

To evaluate the impact of background noise removal on post-stack seismic data, we performed stacking analyses on data before and after denoising, as shown in Figure 13. To ensure accurate velocity parameters for stacking sections, velocity picking was first conducted on the velocity spectrum generated from the denoised data, and the obtained velocity picks were subsequently applied to both the original and denoised datasets during stacking. Figure 13a shows the stacked section after denoising, while Figure 13b presents the stacked section derived from the original data. In the red rectangular region on the left side of Figure 13 (trace range 1270–1900), the fold ranges from 181 to 340. Due to the relatively high fold coverage, this region exhibits strong noise resistance, and thus the difference in SNR between pre- and post-denoising stacked results is not significant. In contrast, in the red rectangular region on the right side (trace range 2300–2500), the fold is only 40–116, resulting in weaker noise suppression capability. As shown in this region, the stacked section after denoising exhibits a markedly higher SNR compared with that of the original data.

### 4.2. Limitations and Future Work

It should be noted that the “clean reference data” used in this study consist of field shot gathers with relatively high signal-to-noise ratios rather than strictly noise-free seismic data. Consequently, residual background noise in the reference data may introduce a discrepancy between the training targets and the ideal noise-free signals, potentially affecting model training and quantitative evaluation. In addition, the SJR metric adopted in this study is primarily used to evaluate the naturalness and global continuity of the denoised results after reassembling individually processed seismic slices. The global mean amplitude difference used in its calculation provides an overall measure of amplitude consistency among different data blocks. However, this global statistical formulation may be influenced by strong reflection events, causing high-amplitude signals to receive disproportionate weight in the SJR calculation and potentially limiting its ability to characterize local continuity and naturalness at stitching boundaries. Therefore, SJR is more appropriate for assessing the overall consistency of reassembled denoising results, while its capability to characterize subtle discontinuities and localized stitching artifacts remains limited. Nevertheless, since all comparison methods were evaluated using the same criterion, the SJR results still provide effective discrimination of differences in stitching continuity among the evaluated methods. Moreover, the proposed method consistently achieved higher SJR values under different experimental conditions, indicating that it can effectively reduce amplitude inconsistencies and stitching artifacts introduced by independent slice-wise processing, thereby producing more natural and globally continuous denoised seismic data.

Furthermore, although the above experiments included a –10 dB noise level, which is outside the SNR range used for training, and the trained model was further applied to field seismic data contaminated by real-world interference from the same survey area, demonstrating a certain degree of SNR extrapolation capability and adaptability to cross-distribution data, the current experimental design provides only limited coverage of testing scenarios outside the training distribution. Therefore, it is insufficient to systematically characterize the performance variations of the proposed model under more substantial distribution shifts. In addition, the field data currently considered in this study are primarily collected from regions in northwestern and southwestern China. The coverage of different geological settings, noise types, and acquisition geometries remains limited. Consequently, the generalization capability of the proposed model across a broader range of geological environments and acquisition conditions cannot yet be fully validated. Due to the limited availability of field datasets and the practical constraints associated with data acquisition, cross-validation across additional regions and acquisition conditions has not been conducted in the present study.

Future research will focus on collecting field seismic noise and effective seismic data from different geographical regions, geological settings, and acquisition conditions, while also investigating the effects of different reference-data construction strategies on model training and evaluation. Based on these datasets, systematic experiments across regions, SNR levels, noise types, and acquisition conditions will be conducted to further evaluate the generalization and extrapolation capabilities of the proposed model under complex data distribution shifts. In addition, the influence of different distributional factors on denoising performance and global seismic continuity will be investigated. Furthermore, local moving-window-based SJR calculations and other localized evaluation metrics will be explored to reduce the influence of strong reflection events on global statistical measures, thereby enabling a more detailed assessment of local noise suppression performance. These efforts will provide more comprehensive experimental evidence for the application of the proposed method to a broader range of real-world seismic data processing scenarios.

## 5. Conclusions

This study proposes a seismic data denoising framework, termed DFRSeisNet, based on wavefield-prior regularization. By jointly optimizing global and local denoising tasks, the proposed framework effectively suppresses complex background noise while enhancing the global consistency of the denoised seismic data. Experimental results demonstrate that the proposed method can effectively alleviate the patching artifacts caused by insufficient local contextual information and discontinuities between independently processed data patches in conventional slice-based denoising. The proposed method also exhibits robust denoising performance on both synthetic noisy data and field seismic data contaminated by real-world interference.

From a practical perspective, this study demonstrates that seismic data denoising under complex anthropogenic noise conditions should not focus solely on local noise suppression, but should also account for the global structural consistency of the entire seismic dataset. Therefore, the proposed method shows considerable potential for seismic data preprocessing in complex noise environments, such as urban fringes and areas with intensive human activities, and can provide more stable and reliable input data for subsequent seismic processing tasks, including seismic event identification, velocity analysis, and imaging. Rather than relying solely on enlarging the receptive field or increasing model complexity, incorporating seismic wavefield characteristics to establish explicit constraints between global and local information provides a new perspective for addressing the inherent trade-off between local modeling and global consistency in large-scale seismic data processing.

## Figures and Tables

**Table 1:** Comparison of four denoising methods on the test set under different noise levels. SJR is not applicable to the full-section wavelet filtering result because no patch stitching is involved.

Input SNR (dB)	Wavelet			MSSA-Net			SwinT			DFRSeisnet		
	SNR	SSIM	SJR	SNR	SSIM	SJR	SNR	SSIM	SJR	SNR	SSIM	SJR
−10	−2.394	0.6802	N/A	7.736	0.8955	2.039	12.304	0.9153	2.071	13.549	0.9256	1.419
−5	−0.426	0.7634	N/A	10.151	0.9337	1.714	14.337	0.9429	1.798	15.476	0.9462	1.341
5	6.239	0.8610	N/A	14.413	0.9480	1.444	19.547	0.9558	1.472	20.183	0.9677	1.242
10	10.244	0.8990	N/A	15.822	0.9526	1.399	22.093	0.9620	1.262	23.407	0.9813	1.197

Bold values indicate the best performance among the compared methods.

**Table 2:** Average validation-set SNR, SSIM, and SJR for UNet, SwinT, and RUNet with and without the proposed framework, corresponding to Figure 10.

Metric	UNet		SwinT		RUNet	
	W/o-DFR	W-DFR	W/o-DFR	W-DFR	W/o-DFR	W-DFR
SNR (dB)	16.162	18.146	20.235	22.491	20.952	**23.542**
SSIM	0.9442	0.9535	0.9616	0.9773	0.9665	**0.9814**
SJR	2.104	1.523	2.037	1.424	2.016	**1.381**

W/o-DFR and W-DFR denote configurations without and with the proposed framework, respectively. Bold values indicate the best performance across all configurations.

## Data Availability

The data presented in this study are available on request from the corresponding author due to confidentiality restrictions of the seismic acquisition company. The source code of the proposed DFRSeisnet is openly available at https://github.com/bickpl/DFRSeisnet (accessed on 13 September 2026).

## Abbreviations

The following abbreviations are used in this manuscript:

ADMM	Alternating direction method of multipliers
CNN	Convolutional neural network
CMP	Common-mid-point
CSP	Common-shot-point
DFR	Fluctuation-prior-regularized
F–K	Frequency–wavenumber
GPU	Graphics processing unit
MSE	Mean squared error
MSSA-Net	Multiscale spatial attention network
RMT	Retentive network meets vision transformer
RUNet	RMT-based U-shaped network
SJR	Stitching jump ratio
SNR	Signal-to-noise ratio
SSIM	Structural similarity index
SUAM	Scale uncertainty-aware module
SwinT	Swin Transformer
